# Responsibility, Free Will, and the Concept of Basic Desert

**DOI:** 10.1007/s11098-022-01912-4

**Published:** 2023-01-18

**Authors:** Leonhard Menges

**Affiliations:** Department of Philosophy (GW), Salzburg University, Kapitelgasse 4-6, 5020 Salzburg, Austria

**Keywords:** Responsibility, Basic desert, Skepticism about responsibility, Free will

## Abstract

Many philosophers characterize a particularly important sense of free will and responsibility by referring to basically deserved blame. But what is basically deserved blame? The aim of this paper is to identify the appraisal entailed by basic desert claims. It presents three desiderata for an account of desert appraisals and it argues that important recent theories fail to meet them. Then, the paper presents and defends a promising alternative. The basic idea is that claims about basically deserved blame entail that the targets have forfeited their claims that others not blame them and that there is positive reason to blame them. The paper shows how this view frames the discussion about skepticism about free will and responsibility.

## Introduction

Many philosophers characterize a particularly important sense of responsibility by referring to basically deserved blame and praise. Derk Pereboom, for example, famously says that for

an agent to be *morally responsible for an action in the basic desert sense* is for the action to be attributable to her in such a way that if she was sensitive to its being morally wrong, she would deserve to be blamed or punished in a way that she would experience as painful or harmful, and if she was sensitive to its being morally exemplary, she would deserve to be praised or rewarded in a way that she would experience as pleasurable or beneficial. The desert at issue is basic in the sense that the agent, to be morally responsible, would deserve such blame or punishment, praise or reward, just by virtue of having performed the action with sensitivity to its moral status, and not, for example, by virtue of consequentialist or contractualist considerations (Pereboom 2012, 11–12; see also 2014, 2).

The expression “basic desert” is philosophical jargon. But it seems plausible that we are familiar with the concept at issue in everyday life. For example, we may want to say that our neighbors deserve praise for helping us move or blame for keeping us awake all night. And we may add that they deserve these responses just because of what they did and how they were when they did it––what they knew, what control they had, and so on––, and not merely because their getting these responses has good consequences or because they have consented to certain norms in advance. In this sense, the desert at issue is basic.^1^

This is a widely accepted minimal characterization of the kind of desert that is relevant for many discussions about responsibility. But can we say something more substantial about it? In this paper I will not aim at a full-blown analysis or definition of basic desert (perhaps there is none; see McKenna 2012, Chap. 5.4; Nelkin 2013, Sect. 3). However, contending that people basically deserve blame or praise seems to entail some kind of evaluation or appraisal of these responses (see, e.g., Feinberg 1970, 56–61; McKenna 2012, Chap. 5.4; 2019a, 257–58; 262). It entails that the speaker take a stand on blaming or praising. But how should we understand this stand? Do basic desert claims entail that it would be good, reasonable, fitting, justified, permissible, obligatory, or something else to blame or praise, or do desert claims involve primitive evaluations? This is the main question of the paper. It is worth stressing again that the aim is *not* to identify necessary and sufficient conditions for its being true that agents basically deserve something. The aim is to clarify the concept of basic desert by analyzing an important entailment of such a claim, namely the appraisal or evaluation that is part of it.

A better understanding of the concept of basic desert is especially important for the free will debate. Many authors who deny or seriously doubt that humans have free will identify free will with the strongest kind of control which is necessary for its being the case that a person is responsible in the basic-desert sense (see Caruso 2018 for an overview). That is, they characterize free will in terms of responsibility and responsibility in terms of basic desert. Thus, a better understanding of basic desert will help us understand the kind of free will skeptics are skeptical about and to make sure that skeptics and defenders are talking about the same thing.^2^

In what follows, I will first present some important desiderata for an account of desert appraisals (Sect. 1) and argue that three representative accounts of basic desert have problems meeting them (Sect. 3). Then, I will develop (Sect. 4) and defend (Sect. 5) a more promising account. Roughly, it says that desert claims entail that those who deserve blame have forfeited their claim against being blamed and that there is reason to blame them.

Some notes before I begin. First, I will use “desert” as an abbreviation of “basic desert”. Second, like most authors in the responsibility debate, I will focus on desert with regard to blame. Third, I will focus on deserved blame for actions. If one can also deserve blame for omissions, attitudes, character traits, or something else, it should be possible to adjust the views I will discuss accordingly. Fourth, an account of desert appraisals is neutral with regard to the question of whether these appraisals are ever true. For example, it may turn out that desert claims entail the appraisal that blame is obligatory and that, in fact, blame is never obligatory. These claims are independent from each other. The paper will only be concerned with the question of how to understand desert appraisals, not with whether they are true.

Finally, the view I will present is compatible with different theories of the nature of blame. Note, however, that Pereboom’s account of responsibility in the basic desert sense is only concerned with blame that is experienced as painful or harmful. Similarly, this paper assumes that the kind of blame that should be at issue in the discussion about skepticism about free will and responsibility is typically *non-trivially harmful* (my own view is slightly more complicated; see Menges 2021). A paradigmatic example is the blamer’s angrily confronting the blamee (see, e.g., McKenna 2013; Fricker 2016; Bagley 2017). Even though I cannot offer an account of when blame’s harm is non-trivial, the rough idea is that it must be severe enough to explain why innocent blamees typically have the standing to demand that the blamers stop. Not everybody will agree that blame is typically non-trivially harmful in this sense. Some argue that to blame is to have an attitude––a belief (e.g., Hieronymi 2004) or emotion (e.g., Wallace 1994, Chaps. 2, 3)––or to modify a relationship (e.g., Scanlon 2008, Chap. 4). And it is not clear that the *harm* of these responses explains why the innocent targets can typically demand that the blamers stop. The paper is neutral with regard to whether this is so. However, if it turns out that these responses are not typically non-trivially harmful, then the paper is not concerned with them. This will be unsatisfying for those who believe that blame is not that harmful. However, in this paper it is more important to clarify the notion of desert that is crucial for the discussion about skepticism than to develop the best account of our everyday notion of blame. And many skeptics are concerned with the desert of responses that are hostile (Rosen, 2015, 67), suffering-causing (Levy 2011, 3), or punishment-like (Strawson 1994, 9; Waller 2011, 2; Caruso and Morris 2017, 841). In order to illuminate this debate, I’m willing to operate with what some may regard as a technical notion of blame and assume that it is typically non-trivially harmful.

## Some desiderata for an account of desert appraisals

Most generally, an account of the appraisal entailed by basic desert claims should explain some important intuitions about what basic desert is and it should help make sense of the debate about free will and responsibility. In what follows, I cannot discuss all intuitions and all aspects of the responsibility debate that an account of desert appraisals should illuminate. Rather, I will focus on three important desiderata that pose problems for famous current accounts of desert that I will discuss in the following section. Let me first present one in more detail that most current accounts of basic desert overlook.

Imagine that Malfoy deserves blame because he knowingly, freely, and without justification stepped on our feet and we blame him for this. Imagine further that we have not done something similar to him or others, our blame is proportionate, and it is not the case that other people have stepped on our feet and we have only singled out Malfoy for blame. Thus, “other things are equal” in this and all following cases I will discuss.^3^ Imagine, now, that Malfoy responds in the following way: “I know that I deserve blame from you, but you owe it to me to stop and I demand that you stop because you are harming me. You should say sorry and compensate me for blaming me!“

There would be something intuitively odd about this response. On the one hand, Malfoy agrees that he deserves blame from us. On the other, he takes himself to be in a position to demand a change in our blame response: he claims that we have a directed obligation towards him, which means that we owe it to him to stop blaming him, express regret or compensate him for being blamed. But, it seems, these two stances can’t go together. Malfoy may reasonably point out that there are *reasons* not to blame him and *ask* us to stop because it harms him. He would be asking us for a favor by doing so. However, if he accepts that he deserves our blame, then he cannot assume that he is in the position to *demand* that we stop blaming. Intuitively, those who deserve to be blamed are, other things being equal, not in the position to legitimately and reasonably demand that they not be blamed, or to demand an expression of regret or compensation from the blamer (see Scanlon 2013, 106 for a similar idea; see Wenar 2020, Sect. 2.2.2 for more on the relevant sense of “demand”).

Some may deny that deserving blame from others and legitimately demanding that they stop are in tension (thanks to a referee for pressing me on this). Imagine that we can choose between harmfully blaming Malfoy and responding in a non-harmful way that plays the same valuable roles that blame would play, such as informing him that we think that his conduct was wrong, making clear that it impaired our relationship, providing him reason to act better in the future, and so on. Some may think that Malfoy can reasonably agree that he deserves our blame and, at the same time, reasonably demand from us that we not blame him and respond in the non-harmful way instead. If this is so, then those who deserve our blame can sometimes reasonably demand that we stop.^4^

Perhaps we have reached a clash of intuitions. To some, it seems incoherent to say that, other things being equal, *S* deserves a harmful response by *P* and that *P* has a directed obligation towards *S* not to respond in this way––such that *S* can reasonably demand from *P* that *P* not do it––regardless of whether alternative responses are available. If this intuition is basic and some do not share it, then some of the arguments against prominent accounts of desert that I will discuss below will not convince everyone. This is an unfortunate dialectical situation, but I fear that we cannot always avoid such situations. The discussion would not be pointless, though. For we would learn that proponents of the views I will discuss below are committed to denying the claim at issue. This would be an interesting conclusion. Moreover, there are ways to back up the claim, not in the sense that there are knock-down arguments for it, but in the sense that it fits well with some ideas that are widely accepted and that many find plausible.

Consider, first, discussions about retributive justice and the claim that guilty criminals deserve punishment. In this debate, a view that is sometimes called negative retributivism says that if agents are guilty wrongdoers and deserve punishment, then they have lost their right not to be punished by justified authorities (for an overview see Walen 2021, Sect. 3.3 and 4.1.3). Now add the independently plausible idea that our right not to be punished corresponds to our being in the position to reasonably demand that others not punish us. Combining this with negative retributivism implies that if there are guilty criminals who deserve punishment, then they cannot demand from justified authorities that they not punish them. This would be so even if there is a non-harmful alternative that plays the same valuable roles that punishment plays. Thus, some standard accounts of punishment rely on the claim at issue, namely: if people deserve some harmful response from others, then they cannot reasonably demand from them not to respond in this way.^5^

Second, consider the intuitively plausible idea that if people deserve our blame for some wrongdoing, then they cannot, other things being equal, reasonably blame us for blaming them for the wrongdoing. Roughly, if Voldemort deserves our blame and we blame him, then there would be something inappropriate about his blaming us for our blame. I take this to be independently plausible. Now, a prominent view on the nature of demands implies that those who think that people who deserve our blame can sometimes reasonably demand from us that we not blame them come in conflict with the idea. The view says that there is a close connection between legitimately demanding something from others and legitimately blaming them if they do not do it. As Gary Watson puts it, to “demand certain behavior of an agent is to lay it down that unless the agent so behaves she will be liable to certain adverse or unwelcome treatment” (Watson 1996, 275). This view suggests that if people can reasonably demand from us that we not blame them, then they can, other things being equal, reasonably blame us if we do it. Now combine this with the idea that people can sometimes deserve our blame and reasonably demand from us that we not do it. Then there are cases in which Malfoy deserves our blame and, at the same time, he can legitimately blame us for blaming him. This is, I think, implausible, which puts pressure on the idea that people can sometimes deserve our blame and reasonably demand that we not blame them.

Let me stress again that these are not knock-down arguments. One could, for example, reply that what is true for deserved punishment is not true for deserved blame or that Watson’s picture of demands and blame is inaccurate.^6^ But in what follows, I will leave these worries aside and work with the following assumption: other things being equal, if agents deserve our blame, then we do not have a directed obligation towards them not to blame them such that they cannot reasonably demand from us that we not blame them or that we express regret or compensate them when we do blame them. The first desideratum for an account of the approval entailed by desert is that it should make sense of this.^7^

A second desideratum for an account of desert appraisals is that desert claims entail that at least *something* positive can be said in favor of blaming those who deserve it (see, e.g., Feinberg 1970, 59–60; McKenna 2019a, 260–61). This positive appraisal does not say that blaming is all-things-considered best or the thing to do. For it makes sense to say that people deserve blame but we should not blame them, perhaps because we should focus on something more important. But it would be odd to say to Malfoy: “You deserve blame for stepping on my foot, but I absolutely don’t approve of and I see absolutely nothing positive about blaming you”.

This intuition can be bolstered by comparing cross-world scenarios (see McKenna 2019a, 263, 269). Consider a world in which nobody blames Malfoy for stepping on our feet. Now consider one which, while being otherwise as similar to the first world as possible, differs in the respect that we do blame him––in the right way and so on––for stepping on our feet. Claiming that Malfoy deserves blame entails that there is some normatively relevant difference between the two worlds. An account of the appraisal entailed by desert should make sense of this.

Finally, an account of desert appraisals should help make sense of what skeptics about and defenders of free will and responsibility disagree about. Again: many authors characterize free will in terms of basic desert. Therefore, a philosophically acceptable account of this notion should be such that those skeptics who accept a desert characterization of free will can reasonably say that they are skeptical about the kind of free will characterized by this account. And those who defend that some humans have the freedom necessary for basically deserved blame or that some humans are responsible in the basic desert sense should also be in the position to accept this account of basic desert.

To sum up, an account of desert appraisals should explain, among other things, that, other things being equal, those who deserve blame are not in the position to demand that the blamers stop, express regret, or compensate them, that desert claims entail some kind of approval, and it should make sense of what skeptics about and defenders of free will and responsibility disagree about.

## Three views on desert appraisals

In what follows I will argue that three recent and representative accounts of desert appraisals fail to meet at least one of the desiderata identified in the previous section.

### Noninstrumentally good harmful blame

A prominent view says that basic desert claims entail the appraisal that there is something noninstrumentally good about harmfully blaming those who deserve to be blamed. Michael McKenna develops an elaborate version of this idea (see also Carlsson 2017; Berman online first, Sect. 3). He discusses two theses about desert (D1 and D2) and then opts for

D3: Because it is noninstrumentally good to harm by blaming one who is blameworthy for a morally wrong act (where the harms in blaming are limited just to those identified on the conversational theory), there is a reason that favors doing so (McKenna 2019a, 273).

Some clarifications seem appropriate. First, even though McKenna pursues a broader goal than this paper, one of his primary aims is to identify the kind of evaluation or appraisal that is entailed when we say that a person deserves blame. On the way, he makes normative claims about the justification of blame in terms of desert (e.g., 2019a, Sect. 7) and he embeds his main ideas in an account of what makes people deserve a response (the desert base) and of the deserved response (blame) (e.g., McKenna 2019a, Sects. 4, 5). However, he explicitly presents the sort of view he is mostly concerned with as reporting “the kind of appraisal––deontological or axiological––a claim of desert is” (McKenna 2019a, 261–62). He adds that we should think of claims like D1, D2, and D3 as saying what is “*entailed by* a certain desert thesis rather than identifying it or giving its (complete) meaning” (McKenna 2019a, 262 italics in original). Therefore, we can reasonably understand D3 as implying McKenna’s answer to our main question of how to understand the appraisal entailed by basic desert claims. D3 suggests: When we say that agents basically deserve our blame, then this entails that it is noninstrumentally good to harm by blaming them (where the harms in blaming are limited just to those identified on McKenna’s conversational theory), and that, therefore, there is a reason that favors our blaming them (thanks to a referee for pressing me on this).^8^

Second, it is worth stressing that McKenna does not contend that any kind of harm would be good. According to him, the *harm of blaming* is sometimes noninstrumentally good and he spells out blame in terms of his conversational account, according to which the harm of blame is relatively mild (McKenna 2012; 2013; 2019a, Sect. 6.1; see also Bennett 2002). Similarly, he does not say that the harm considered on its own is noninstrumentally good. On his view, the harm is only noninstrumentally *good insofar it is part of a noninstrumentally valuable whole*, namely the kind of conversation that is at the heart of his account of responsibility (McKenna 2019a, 277–78). However, he does not only say that the harm of blaming is sometimes good because it has valuable effects or that it is acceptable as a side-effect of blaming the blameworthy. On his view, harming by blaming the blameworthy is noninstrumentally good: “To be clear, what is it that is a candidate for a noninstrumental good? It is the harm itself as it contributes to the blaming activity, an activity that is in response to, and so is extrinsically dependent upon, an antecedent blameworthy act” (McKenna 2019a, 276). Thus, we can reasonably understand McKenna as suggesting that to claim that agents basically deserve blame entails that it is noninstrumentally good to harm by blaming them, which is why there is reason to do it. Let us see whether this view meets the desiderata identified above.

One desideratum says that an account of basic desert appraisals should explain why agents who agree that they basically deserve blame cannot, other things being equal, reasonably demand that the blamers stop, express regret, or compensate them. McKenna’s view says that basic desert claims entail that harmful blame is noninstrumentally good and there is reason that favors blaming. However, something can be noninstrumentally good such that there is a reason that favors our doing it and it may still be true that people can reasonably demand that we stop. For example, it may be noninstrumentally good for you and from the point of view of the universe that you and Dumbledore become good friends such that there is reason that favors your becoming friends with Dumbledore. Imagine that I can magically make it the case that you and Dumbledore become good friends. You surely have the standing to demand from me that I don’t do it if you do not want to be his friend. If I, nonetheless, magically make you become friends, I owe you an expression of regret, an apology, and perhaps (symbolic) compensation.

Now, if the appraisal entailed by desert is to be understood in terms of noninstrumental goodness and positive reasons, as McKenna claims it should, then the same is true for deserved blame. Even if it is noninstrumentally good such that there is a reason in favor of blaming Malfoy for stepping on our feet, this does not rule out that he can reasonably demand that we stop. However, people who deserve blame cannot reasonably respond in this way. They may sensibly ask the blamers to stop and point to the reason that blame is harmful. But they cannot demand from the blamers to stop in the way in which you can demand that I do not make it the case that Dumbledore and you become good friends. Thus, McKenna’s account of the appraisal entailed by desert does not meet the first desideratum.

Second, an account of desert appraisals should make sense of the idea that desert claims entail some kind of approval. McKenna’s view easily meets this requirement. It says that blaming those who deserve it is noninstrumentally good such that there is a reason in favor of blaming them. This is, obviously, an approval.

However, McKenna’s account has a surprising and, I believe, unhelpful implication for the debate about skepticism. On purely conceptual grounds, it implies that a seemingly acceptable combination of widely held views on metaphysical and normative matters is incoherent, namely non-skepticism about responsibility in the basic desert sense on the one hand and the view that harm is never noninstrumentally good on the other.

Recall that the discussion about responsibility in the basic desert sense relies on the following assumption:

(i) If agents are responsible in the basic desert sense for bad actions, then they basically deserve blame for them.

The question of this paper is how to understand the appraisal entailed by the claim that someone basically deserves blame. McKenna’s answer is:

(ii) If agents basically deserve blame, then it is noninstrumentally good to harm them by blaming them (proportionally and in accordance with the conversational account of blame) and, therefore, there is reason to blame them.

Combining (i) and (ii) delivers:

(C1) If agents are responsible in the basic desert sense for bad actions, then it is noninstrumentally good to harm them by blaming them (proportionally and in accordance with the conversational account of blame) and, therefore, there is reason to blame them.

Now non-skeptics about responsibility in the basic desert sense say that some humans are responsible in this sense:

(iii) If non-skepticism about responsibility in the basic desert sense is true, then some humans are responsible in the basic desert sense.

Combining (C1) with (iii) yields:

(C2) If non-skepticism about responsibility in the basic desert sense is true, then harming humans in specific ways (by blaming them) is sometimes (when they are responsible for bad actions in the basic desert sense) noninstrumentally good.

However, there are many authors who hold the following independent view (e.g., Parfit 2011, Sect. 39; Scanlon 2013; Smith 2019; List in List, Caruso, and Clark 2020):

(iv) Harming humans is never noninstrumentally good.

According to (iv), harming may be instrumentally good (perhaps by deterring future wrongdoers) or acceptable as an unwelcome side-effect (perhaps in cases of self-defense). But it says that harm cannot be *non*instrumentally good. Now, (C2) and (iv) imply.

(C3) Non-skepticism about responsibility in the basic desert sense is false.

Thus, McKenna’s account of basic desert––premise (ii)–– implies that those who hold the independent normative view that harm is never noninstrumentally good are skeptics about responsibility: if you believe that harm is never noninstrumentally good, then you can’t say that some humans are responsible in the basic desert sense. For this would commit you to accepting that harming by blaming the blameworthy is noninstrumentally good.^9^

In other words, McKenna’s account of basic desert makes skepticism about responsibility the default position for those who believe that harm is *never* noninstrumentally good. However, skepticism is typically presented and plausibly thought of as the conclusion of substantial arguments about luck, determinism, control, or God––not as the view one starts with as soon as one understands the concepts at issue and holds the independent view that harming is never noninstrumentally good.

All this does not aim at showing that McKenna’s view is false. However, I take it to be a vice of this account that it frames the debate about skepticism in such a way. If there is an otherwise at least equally plausible alternative which leaves conceptual space for being non-skeptical about responsibility in the basic desert sense and skeptical about the noninstrumental goodness of harm, then there is initial reason to accept this view rather than McKenna’s.

Thus, McKenna’s account of desert appraisals has problems meeting two of the three desiderata.

### Fitting blame

Another famous view characterizes desert appraisals in terms of fittingness. According to this account, saying that agents deserve blame entails that blaming them would be fitting similar to the sense in which it would be fitting to be amused by a funny joke (see, e.g., King 2012; Shoemaker 2015, 220–23 who puts it in terms of fitting anger, rather than blame; Nelkin 2016). Typically, fittingness appraisals are construed as non-moral appraisals that are independent of claims about goodness. To illustrate, the relevant sense of fittingness is supposed to be such that it can be fitting to laugh at (Nelkin 2016, 178) or be amused by (Shoemaker 2018, 82) immoral, say, racist jokes. As Dana Nelkin presents her view in most detail, I will focus on it (Nelkin discusses desert in 2013; 2016; 2019a; 2019b; 2019c; the following focuses on 2016).

Nelkin contends that people can basically deserve blame without there being a positive reason to blame them. She argues that fittingness claims in general do not entail claims about positive reasons. One of her examples is that it is fitting of an artifact to fulfill its function. But “the fact that it would fulfill a fountain’s function to be filled with water and turned on, say, does not by itself provide reason to do so in a drought, for example” (Nelkin 2016, 178). Nelkin then argues that there is a more complex connection between desert and reasons that she spells out in her Conditional Reason account: “(CR) X’s being deserving of sanction is a conditional reason to sanction X” (Nelkin 2016, 179). That is, desert alone does not provide a reason to sanction. But if an agent deserves a sanction and certain conditions are fulfilled, then the desert provides a reason to sanction the agent. What are the conditions? Nelkin makes only one suggestion:

Suppose that you are in a position in which you have no choice but to inflict harm and you can harm someone deserving of it or harm someone else. This may give you a reason to harm the person who is deserving rather than others (Nelkin 2016, 179; see also her view on punishment 2019b, 435).

This paper is concerned with harmful blame and not with harm simpliciter or sanctions in general. However, it seems very plausible from the context of the debate that Nelkin would also apply CR to harmful blame (see also Nelkin 2019c, 189). Thus, where McKenna analyzes the desert appraisal in terms of noninstrumental goodness and positive reasons, Nelkin analyzes it in terms of fittingness and conditional reasons. How does this view deal with the three desiderata?

First, Nelkin’s account also cannot explain why those who deserve blame are not in the position to demand that blamers stop, express regret, or offer them compensation for being blamed. To see this, consider Nelkin’s analogy between blame and laughter. She assumes (Nelkin 2016, 178) that laughing at a racist joke can be fitting. However, this does not rule out that members of the racialized group whom the joke is about can demand from those who laugh an expression of regret or (symbolic) compensation because it caused them pain. Indeed, it seems very plausible that such a demand is often appropriate. If basic desert should be understood analogously to the fittingness of laughter, as Nelkin claims it should, then the same holds for deserved blame. Its being fitting to blame certain agents and its being such that if one has to blame someone then there is reason to fittingly blame do not rule out that these agents can appropriately demand expressions of regret or compensation for being blamed. However, if a person deserves blame, then she cannot reasonably demand this. Thus, Nelkin’s view does not meet the first desideratum.

Nelkin’s fittingness account also has problems with the approval aspect of desert appraisals. That is, she cannot make sense of the oddity of your saying to Malfoy: “You deserve blame for stepping on my foot, but I absolutely don’t approve of and I see absolutely nothing positive about blaming you”. Her view says that if we have to harmfully blame people, we have a reason to blame those who deserve it. However, the view cannot make sense of the idea that if agents deserve blame in cases in which we do not have to blame anyone, then something can be said in favor of blaming.

Nelkin is well aware of this objection (Nelkin 2016, 178). However, she contends that desert appraisals are fittingness claims and she argues that fittingness claims do not entail claims about positive reasons. As a reply, there seem to be important differences between fittingness claims as she understands them and basic desert claims. Take her example that it is fitting for a fountain to be filled with water and turned on. Are those who accept it committed to there being a normatively relevant difference between a world in which the fountain is filled with water and turned on and a world in which it is not and where everything else is, as far as possible, equal? Intuitively, they are not. However, those who claim that Malfoy deserves blame seem to be committed to saying that there is a normatively relevant difference between a world in which he is and one in which he is not blamed and where everything else is, as far as possible, equal. Understanding basic desert in terms of mere fittingness cannot make sense of this. This is a problem for the fittingness account.

To sum up, the fittingness account does not meet the first and second desideratum for an account of desert appraisals. However, it does not seem to have problems meeting the third desideratum. It does not rule out otherwise plausible views and it says what skeptics and defenders disagree about. According to this view, skeptics say that there is good reason to think that because humans lack a certain kind of control it is never fitting to blame them, while defenders contend that some humans do have the relevant kind of control.

### Justified blame

A third famous view says that desert claims entail the appraisal that blame is justified or warranted. Gregg D. Caruso and Stephen G. Morris present such an account. They call the kind of responsibility which is at issue in debates about skepticism retributivist desert moral responsibility: “The question of retributivist desert moral responsibility is […] about whether it would ever be justified or warranted to blame or punish a wrongdoer on purely backward-looking, non-consequentialist grounds” (Caruso and Morris 2017, 842). Let us put punishment aside and focus on blame. Citing Sofia Jeppsson, Caruso and Morris specify that contending that an agent is responsible in the relevant sense entails that “*in the absence of sufficiently strong counter-veiling reasons*, blaming her is justified if her action was wrong” (Jeppsson 2016, 683 italics in original; Caruso and Morris 2017, 842). Thus, according to this view, claiming that Malfoy is responsible for stepping on our feet entails that he deserves blame for it, which implies that blaming him is justified as long as there are no outweighing reasons against blaming him.

This account has no problems with the second and third desideratum. It says that desert claims entail that blame is justified, as long as there are no sufficiently strong reasons against blaming, which is a kind of approval. The view also frames the debate about skepticism in clear ways. According to skeptics, there is reason to doubt that blaming humans is ever justified by purely backward-looking considerations because they lack a kind of control, while defenders of responsibility claim that it sometimes is justified by these considerations. And the view does not rule out otherwise plausible philosophical theses.

However, this view also does not meet the first desideratum. It can be justified or warranted for us to do something and the affected people can demand an expression of regret or compensation for our doing it. Take Joel Feinberg’s famous cabin case:

Suppose that you are on a back-packing trip in the high mountain country when an unanticipated blizzard strikes the area with such ferocity that your life is imperiled. Fortunately, you stumble onto an unoccupied cabin, locked and boarded up for the winter, clearly somebody else’s private property. You smash in a window, enter, and huddle in a corner for three days until the storm abates. During this period you help yourself to your unknown benefactor’s food supply and burn his wooden furniture in the fireplace to keep warm (Feinberg 1978, 102).

It is very plausible that you are justified in doing this. However, you surely owe the owner compensation for what you have done and, plausibly, an expression of regret for your having to do it. Thus, saying that you are justified in doing something is compatible with agreeing that you owe the affected people compensation and expressions of regret for it. If desert claims are to be understood in terms of justified blame, as Caruso, Morris, and Jeppsson claim, then it can make sense for Malfoy to agree that he deserves blame and to demand an expression of regret or compensation for being blamed. However, this does not make sense. Therefore, the justified-blame account has the same problem with the first desideratum that McKenna’s and Nelkin’s views have.

## The claim forfeiture view on basic desert

Here is a straightforward account of the appraisal entailed by basic desert claims, which I will call the *Claim Forfeiture View on Basic Desert* or, for short,

CFD: Claiming that agents *S* basically deserve to be blamed because of *x* (which is some fact about what *S* did and how *S* were when *S* did it) entails that *x* makes it the case that

 (1) other things being equal, *S* have forfeited their claims against others that they not blame them for *x* and

 (2) there is reason to blame *S* for *x*.^10^

CFD starts from the assumption that blaming people typically non-trivially harms them (see Sect. 1). Therefore, as long as people are not blameworthy, they have a claim against others that they refrain from blaming them. To blame the innocent by, for example, angrily confronting them, would, other things being equal, violate their claims and wrong them. Moreover, there is typically no reason to blame the innocent. Blaming your neighbors even though they did not do anything objectionable is, typically, pointless.^11^ However, if people are blameworthy for an objectionable action and deserve blame for it, then this explains two things: first, that they forfeit their claim not to be blamed such that blaming them would not infringe one of their claims and would not wrong them; second, that there is reason to blame them.

Note that like McKenna’s and Nelkin’s view, CFD has two parts. McKenna makes sense of basic desert claims in terms of noninstrumental goodness and positive reasons, Nelkin in terms of fittingness and conditional reasons, CFD in terms of claim-forfeiture and positive reasons to blame. However, in contrast to the three accounts discussed above, CFD meets all the desiderata presented in Sect. 2.

### The blamees’ position to make demands

Claim (1) of CFD makes sense of the idea that, other things being equal, those who basically deserve to be blamed cannot appropriately demand not to be blamed, an expression of regret, or compensation. Part of what it is to have a claim against others is that if they infringe this claim then one is in a special position to respond in some of these ways (see, e.g., Darwall 2006, 18–19; Wallace 2019, 6–9). To illustrate, when Malfoy steps on your foot, then you are in a special position to demand that he stop, say sorry, and so on. This is so because he violates a claim that you have against him. When you break into the cabin to save your life you do not violate the owner’s claim, but you permissibly infringe it. Then, they cannot demand that you stop, but they can demand compensation and, plausibly, an expression of regret (see Feinberg 1978, 102).

According to CFD, if people deserve blame and other things are equal, then they forfeit their claim against others––such as their victims––that they refrain from blaming them. One would not even infringe their claim by blaming them because they do not have it. That they did something bad and the way they were when they did it erased their claim against being blamed; their conduct negated that certain others have a directed obligation against them that they not blame them. Then, it would be inappropriate of the blamees to demand that one stop blaming, express regret, or provide compensation, and they cannot reasonably complain that blaming them infringes their claims.

Note that CFD is compatible with the idea that there are *reasons* against blaming those who deserve it or for regretting that one blamed them or for compensating them for being blamed. The blameworthy may point to these reasons and *ask* the blamers to stop. CFD only says that those who deserve blame do not have a *claim* against certain others that they respond in these ways. Correspondingly, those who deserve blame do not have the standing to demand from the blamers that they stop blaming, express regret, or compensate them. And the blamers do not owe it the blamees to respond in these ways.

Thus, CFD elegantly explains why those who deserve blame are, other things being equal, not in the position to make certain demands.

### The approval aspect of desert claims

According to CFD, desert claims entail that there is reason to blame those who deserve it. This is a positive normative appraisal of blaming. It does not imply that there is sufficient or overwhelming reason to blame. For it still makes sense to say that people deserve to be blamed but there is most reason not to blame them, perhaps because one should concentrate on something more important. However, if they deserve to be blamed, then *something* can be said in favor of blaming them.

CFD is compatible with but not committed to the claim that there is noninstrumental reason to harm those who deserve blame (thanks to a referee for pressing me on this). People with strong retributivist intuitions may want to say this and they can easily combine it with CFD. However, one can accept CFD, believe that some people deserve blame, and deny that there is ever noninstrumental reason to harm people. This is so because one can say that there is noninstrumental reason to respond in a certain way and that this response harms others, without having to say that there is noninstrumental reason to harm them. The harm would only be a side effect that is not directly supported by any noninstrumental reason. For example, we sometimes have noninstrumental reason to tell our friends the truth. Then, there are situations in which Hermione has noninstrumental reason to tell Ron that he is envious. This would harm Ron. It does not follow from these claims that Hermione has noninstrumental reason to harm Ron. One can coherently add that there is nothing that speaks directly in favor of harming Ron. Similarly, we can hold that we have noninstrumental reason to blame Malfoy for stepping on our feet, that it would harm him, and we can deny that there is ever noninstrumental reason to harm people.

Note that CFD is silent with regard to the questions of whether and why facts about what the agents did and how they were when they did it ground reasons to blame. This is how it should be, because we need to distinguish between the conceptual question of what basic desert claims entail and the normative question of what reason there actually is to blame people. CFD is an answer to the conceptual question, not to the normative. Thus, demanding of CFD to provide an account of the reasons to blame would express a misunderstanding of what CFD is trying to achieve. However, CFD can be complemented by such a normative account. Let me briefly sketch one.

A plausible and widely accepted picture says that blame plays important communicative roles (see, e.g., Watson 1987; Bennett 2002; McKenna 2013; Macnamara 2015; Shoemaker 2015, Chap. 3; Fricker 2016; Bagley 2017; Mason 2019, Chap. 5; McGeer 2019; Sliwa 2019; Wang 2021). If we have been wronged, we seem to have reason to make clear that we do not accept this treatment. This is not simply a reason to vent anger. Rather, it is a reason to communicate to ourselves, the blameworthy, or to third parties that it was wrong to treat us this way. Sometimes, we also have reason to communicate non-acceptance when we are not the direct victims. If we are committed to moral norms and values, then it is often reasonable to make clear that we do not accept their being disrespected and violated. Blaming the blameworthy communicates this.

Moreover, we often have reason to communicate that we want or demand that the blameworthy acknowledge their fault, dissociate from it, and, perhaps, offer compensation. Ideally, the blameworthy would publicly and sincerely accept that they have wronged someone, disrespected a moral value, or violated a moral norm, that they should not have done it, and promise that they will not do it again. Blaming the blameworthy communicates that we want or demand that they acknowledge their fault in such a way.

Thus, it is quite plausible that people’s objectionable behavior combined with some facts about how the agents were when they behaved this way––such as what they knew and what control they had––can ground reasons to blame them. Combining CFD with a normative account along these lines can, therefore, make sense of the idea that there is reason to blame those who deserve it. But note again that this sketch of a normative account is not part of CFD, which is a conceptual, not a normative thesis. Thus, CFD can be true even if this normative view is misguided.

### Framing the debate about skepticism

CFD leaves enough conceptual room for a plausible wide range of metaphysical and normative views. According to CFD, contending that people deserve blame entails that we have reason to blame them, which typically causes harm, but would not infringe their claims. This view is not committed to saying that harm is noninstrumentally good. This is so because, generally, saying that we have reason to do something that harms others but does not infringe their claims does not imply that harming them would be noninstrumentally good. Take the example of truth-telling again. That Hermione has noninstrumental reason to tell Ron the truth, which would harm him, does not imply that there would be something noninstrumentally good about harming Ron. Or imagine that you have locked my bike with your lock without having asked me, I have to go home quickly, and I cannot find you. Normally, you have a claim against my picking your bike lock, but in this situation I would not infringe any claim by doing it. It surely does not follow that harming you by picking your bike lock is noninstrumentally good. Analogously, contending that we have reason to blame people which would cause harm but would not infringe their claim does not imply that the harm of blame would be noninstrumentally good. Thus, CFD allows combining the view that some people are responsible and deserve blame with the view that harming is never noninstrumentally good.

Moreover CFD nicely makes sense of what skeptics about and defenders of free will and responsibility disagree about. According to CFD, claiming that people are responsible for some bad action in the basic-desert sense entails that they have forfeited their claim not to be blamed for it and that there is reason to blame them for it. The skeptical starting point would then be the plausible idea that forfeiting the claim not to be blamed for some action presupposes that this action is the result of some kind of agency. For example, when I step on your foot simply because I was pushed, it seems that I still have a claim against you that you not blame me for stepping on your foot. Skeptics can argue that humans never have the kind of agency which is necessary for forfeiting their claim against others (just because of what they did and how they were when they did it) that they not blame them. According to this view, part (1) of CFD is never true and blaming people always infringes their claims.

To illustrate, take the manipulation argument for incompatibilism about responsibility and determinism (see, e.g., Pereboom 2014, Chap. 4; Mele 2019). It starts with a science fiction scenario in which neuroscientists manipulate an agent. Because of the manipulation, the agent is determined to have a certain kind of agency––a reason-responsive mechanism, certain first- and second-order desires, a specific deep self or quality of will––and a bad action is the result of this agency. According to CFD, the manipulation argument should, then, be as follows: first, if agents are, because of a manipulation, determined to have a certain agency that results in a bad action then they do not, thereby, forfeit their claims against being blamed for the action. Second, there is no difference between being determined because of manipulation and because of determinism that is relevant for claim forfeiture. Therefore, if agents are, because of determinism, determined to have a certain agency that results in a bad action then they do not, thereby, forfeit their claims against being blamed for the action.

Compatibilists about responsibility and determinism would reply that one of the premises is false. The hard-line reply attacks the first premise (see, e.g., McKenna 2014). According to CFD, it should say that agents who are determined to have a certain agency that results in a bad action because they are manipulated can, thereby, forfeit their claims against being blamed for the action. On this view, those who are manipulated to do something bad can, thereby, lose their standing to demand from blamers that they stop blaming them for the action. And if they are blamed then they cannot reasonably demand an expression of regret or (symbolic) compensation.

CFD does not tell us whether the first premise of the manipulation argument is true or false. However, it provides a clear and new way to frame the discussion about it. On this view, incompatibilists need to argue that manipulation rules out claim forfeiture and compatibilists need to show that this is not so. Accordingly, future discussions would profit from exploring the nature of claims and claim forfeiture more generally.

What is true for discussing the manipulation argument in particular holds for discussing skepticism about free will and responsibility more generally. According to CFD, skeptics about responsibility should argue that humans never have the kind of agency or knowledge which is necessary for forfeiting their claim against others (just because of what they did and how they were when they did it) that they not blame them. Defenders of responsibility should reply that some humans have this kind of agency and knowledge. According to this view, not all instances of blame constitute a claim infringement. This is a new and potentially fruitful way of framing the discussion about skepticism.

To sum up, CFD has no problems meeting the desiderata for a plausible account of desert appraisals presented in Sect. 2. Thus, it has important advantages over the three standard alternatives discussed above.

## In defense of the Claim Forfeiture View

### The too weak objection

McKenna discusses the view that basic desert claims entail the appraisal that blame is permissible, in the sense that “one would do no wrong to blame” (McKenna 2019a, 260). But permissibility does “not provide a positive reason *to* blame” (McKenna 2019a, 260 italics in original). He argues that, according to this account, it can be true that an agent deserves to be blamed and that one always has most reason not to blame humans because it is so harmful. McKenna takes this appraisal to be too weak. He says about the view:

But if blaming involves harming, it seems one should *not* blame unless she has a good reason to do so; the harming would itself seem to offer a reason *against* blaming, even if it is permissible to harm. So a culpable person’s deserving blame would never outweigh a would-be blamer’s reasons to refrain from blaming. That cannot be right (McKenna 2019a, 260 italics in original).

A modified version of this objection hits CFD. In contrast to the view discussed by McKenna, CFD says that if agents deserve to be blamed, then there is reason to blame them. But CFD does not say anything about the weight of these reasons. Thus, CFD does not rule out that the harm-based reasons against blaming always outweigh the reasons for blaming.

As a first reply, this objection may be based on a blending of conceptual and normative questions. If we want a *justification* of our actual blame practice in terms of desert, then we need to show that the reasons picked out by desert claims sometimes outweigh countervailing reasons. However, it is not obvious that a *conceptual* account of desert needs to do this. It seems plausible that an account of the appraisal entailed by desert claims can leave it open whether there is weightier reason for or against blame.

Some may object that a conceptual account should not leave this question open because the following is incoherent: “Some people basically deserve blame, but as blaming is so harmful, we always have weightier reason not to do it”. I find this claim much less odd than the ones discussed in Sect. 2. But let us grant, for the sake of the argument, that it is incoherent.

Then, proponents of CFD can point to claim (1), which says that, other things being equal, if agents deserve blame, then they have forfeited their claim not to be blamed. They could argue that if the harm-based reasons against blame always outweigh the reasons for blame, then the targets of blame never forfeit the claim not to be blamed. Of course, this view needs to be backed up in more detail. But it seems very plausible that if a response is so harmful that the reasons against it are always weightier than those in favor of it, then this response infringes a claim of the target. The case of torture may be analogous. One may say that if torturing people is so harmful or disrespectful that there is always weightier reason not to torture than to do it, then people never forfeit their claim not to be tortured.

Based on this idea, one can conclude that if the harm-based reasons against blaming are *that* weighty, it follows that humans never deserve to be blamed because they never forfeit their claim not to be blamed. Thereby, one could make sense of the idea that there is something incoherent about saying: “Sometimes people basically deserve blame, but as blaming is so harmful, we always have weightier reason not to do it”. To be clear, I am not assuming that a conceptual account of basic desert needs to make sense of the idea that this claim is incoherent. But if we assume that it should, then CFD has good chances of meeting this challenge.

### The fragmentation objection

One may object that CFD is problematic because it yields a fragmented account of desert appraisals. According to CFD, basic desert claims entail that someone has forfeited a claim against blame and that there is reason to blame. CFD does not show that these entailments are unified. However, the objection says, basic desert claims entail unified appraisals, not fragmented ones.

As a reply, it is not obvious that basic desert claims entail unified appraisals. I don’t know an argument to this conclusion and accounts of basic desert that yield a unified appraisal, like Caruso’s and Morris’, have problems in other respects. Moreover, many important philosophical concepts are not unified. Knowledge is typically analyzed in terms of truth, justification, and other things, responsibility is sometimes analyzed in terms of control and knowledge, and motivating reasons in terms of beliefs and desires. Of course, these accounts may be problematic. But they are surely not problematic because they yield that the analyzed concepts are not unified. Proponents of CFD can reply in the same way to the fragmentation objection. Therefore, I take CFD to be defended.

## Conclusion

CFD makes sense of some strong intuitions about basic desert and leaves room for a plausible variety of positions about the normative status of blame and about responsibility. Moreover, it frames the debate about free will and responsibility in a helpful way. It says that free will is the strongest kind of agency which is necessary for forfeiting one’s claim against others (just because of what one did and how one was when one did it) that they refrain from blaming. Skeptics doubt that humans have this agency, defenders contend that some have it. Moreover, the view makes sense of the importance of the free will debate: if humans lack free will, then our blame practice constitutes a massive claim infringement.^12^

## Data Availability

Not applicable.

## Footnotes

1 Note that basic desert, thus understood, is independent of facts about deterrence. If one thinks that whether people deserve blame is partly a function of whether blame would deter them from future wrongdoings, then one does not operate with the notion of basic desert this paper is concerned with.

2 McKenna suggests that the kind of responsibility that is relevant for free will skepticism does not need to be analyzed in terms of basic desert. However, most skeptics still put their position in terms of basic desert (for discussions see McKenna 2019c; 2019b; Pereboom 2019; Nelkin 2019a).

3 More generally, the other-things-being-equal clause is meant to bracket worries about, e.g., the standing to blame, the proportionality of blame, and the distribution of blame.

4 Here is another possible objection: imagine a situation in which even mildly blaming Malfoy would ruin his life. Can’t he coherently agree that he deserves blame and demand that we stop in this case? I will discuss this point in more detail in Sect. 5.1. The brief answer is that it would probably be false to say that he deserves blame in this case. Nobody deserves a response that would ruin their life because they stepped on someone’s foot. Thus, in this case, Malfoy can reasonably demand that we stop blaming him, but he does not deserve blame.

5 Note that the line of thinking presented here is compatible with the idea that any kind of retributivism is false as a normative view about what people in fact deserve. We only need to assume that it is coherent. Again, the goal of the paper is to understand the notion of desert, not to say whether anyone ever deserves something in the relevant sense.

6 Personally, I have doubts about the claim that legitimate demands go hand-in-hand with legitimate *harmful* blame when people don’t do what we demand from them. See also Macnamara (2013).

7 A potential misunderstanding should be addressed. The notion of basic desert at issue in responsibility scholarship should not be confused with the notion of desert that is at issue in the context of distributive justice. Imagine that the members of Dumbledore’s Army distribute jobs in a fair way (e.g., by throwing a die) and imagine that Parvati gets a particularly unpleasant one. Perhaps, in this context it would make sense (I do not take a stance on this) for her to agree that she deserves the job and demand that they not give it to her. Even if it does make sense, there are many important differences between distributive desert and basic desert. For example, whether someone deserves something in the former sense depends partly on the resources available, while basic desert only depends on what the agents did and how they were (see Smilansky 2006 for this and other differences). Therefore, what is true for the notion of desert that is at issue in the context of distributive justice is not necessarily true for the notion of basic desert that is at issue in responsibility scholarship.

8 Another way to put it is this: According to D3, *S*’s appraisal of *P* is the appraisal entailed by desert claims just in case *S* evaluates harming by blaming *P* as noninstrumentally good (where the harms in blaming are limited just to those identified on McKenna’s conversational theory), which is why there is a reason that favors blaming *P*.

9 Note that McKenna’s view does not imply that authors like Parfit are skeptics about *free will* (thanks to a referee for pressing me on this). Rather, it implies that they are skeptics about *responsibility in the basic desert sense*. One can be skeptical about this kind of responsibility without being skeptical about free will. For example, one can be neutral about free will or even believe that some humans have free will and deny that humans fulfill the knowledge condition for responsibility. McKenna’s view implies that Parfit, Smith, Scanlon, List, and so on would belong to a third group of skeptics about responsibility. This group is not skeptical about the relevant kind of control or knowledge. They would be skeptics just because of their views on the value of harm.

10 Neil Levy’s characterization of responsibility has some similarities with claim (1) of CFD: “Someone who holds that agents can be morally responsible for wrongful acts or omissions […] is committed to holding that such agents no longer deserve the (full) protection of a right to which they would otherwise be entitled: a right against having their interests discounted in consequentialist calculations” (Levy 2011, 3).

11 I say that there is *typically* no reason to blame the innocent because it is easy to imagine a non-typical situation in which there is reason to do so. For example, you would have reason to blame your innocent neighbors if you were going to get a lot of money from a crazy millionaire for doing so.

12 I am very grateful to Hannah Altehenger, Daniele Bruno, Max Kiener, Sebastian Köhler, Stefan Riedener, and Peter Schulte for helpful written comments and discussions. I also want to thank the participants of a video discussion with the philosophers at Lund University in April 2020 and an online audience at the *25. Deutscher Kongress für Philosophie* in September 2021 for comments and suggestions. The paper profited a lot from comments by referees for the *Australasien Journal of Philosophy*, *Synthese*, and especially from detailed and constructive criticism by referees for *Philosophical Studies*. I’m very grateful to the referees. Finally, thanks to Claire Davis for proofreading different versions of the paper. Work on the paper was supported by the Austrian Science Fund (FWF) P34851-G and is part of the research project *The Sense of Responsibility Worth Worrying About*.
